# Phase I study of irinotecan and gefitinib in patients with gefitinib treatment failure for non-small cell lung cancer

**DOI:** 10.1038/bjc.2011.375

**Published:** 2011-09-13

**Authors:** A Horiike, K Kudo, E Miyauchi, F Ohyanagi, K Kasahara, T Horai, M Nishio

**Affiliations:** 1 Thoracic Oncology Center, Cancer Institute Hospital, Japanese Foundation for Cancer Research, 3-8-31 Ariake, Koto-ward, Tokyo 135-8550, Japan; 2 Department of Respiratory Medicine, School of Medicine, Kanazawa University, 13-1 Takara-machi, Kanazawa 920-8641, Japan

**Keywords:** gefitinib, resistance, irinotecan, non-small cell lung cancer

## Abstract

**Background:**

Currently, no effective treatments exist for non-small cell lung cancer (NSCLC) after failure of gefitinib therapy. Pre-clinical studies have demonstrated that gefitinib-resistant NSCLC cells are more sensitive to irinotecan than parental cells, and that combined administration of irinotecan and gefitinib has a synergistic additive effect. We conducted a phase I study to evaluate the combination of irinotecan and gefitinib as a therapeutic option for NSCLC patients with progressive disease (PD) after initial gefitinib treatment.

**Methods:**

Eligibility criteria included histologically confirmed NSCLC, age range of 20–74 years, refractory to or relapsed after gefitinib treatment, one or more previous chemotherapy regimens, Eastern Cooperative Oncology Group performance status 0–2, adequate organ function, and informed consent. Patients were treated with irinotecan on days 1 and 15, and treated daily with gefitinib from day 2 every 4 weeks. The treatment was continued until disease progression. The gefitinib dose was fixed at 250 mg. Irinotecan dosing started at 50 mg m^−2^ and was escalated in patients by 25 mg m^−2^ increments up to a maximum dose of 150 mg m^−2^.

**Results:**

Twenty-seven patients were enrolled: male/female=14/13; median age=60 (45–75); histology, adenocarcinoma/non-adenocarcinoma=25/2; performance status 0–1/2=19/8; previous response to gefitinib, partial response/stable disease/PD=21/2/4. Dose-limiting toxicities were observed in 2 patients at level 3. Maximum tolerated dose was not determined, and the full dose of irinotecan could be combined with the full dose of gefitinib. The disease control rate (DCR) and response rate (RR) were 69.2 and 26.9%, respectively. For 12 patients at level 5 (the recommended phase II dose), the DCR and RR were 75.0% and 41.7%, respectively. The median treatment cycles were 4; median time to treatment failure, 57 days (95% confidence interval (CI), 32–82 days); median overall survival, 244 days (95% CI, 185–303 days); and 1-year survival rate, 32.6%.

**Conclusion:**

The combination of irinotecan and gefitinib was well tolerated and potentially beneficial for NSCLC patients failing initial gefitinib monotherapy.

The epidermal growth factor receptor (EGFR) is a well-established target for anticancer therapy because it is expressed or overexpressed in a variety of tumours, including non-small cell lung cancer (NSCLC) (Rusch *et al*, 1993). Gefitinib is an EGFR tyrosine kinase inhibitor (TKI), and the first targeted drug developed and approved for NSCLC (Fukuoka *et al*, 2003; Kris *et al*, 2003). Various large phase III studies have been performed on unselected previously treated NSCLC patients. In a study performed by Kim *et al* (2008), gefitinib monotherapy resulted in survival that was non-inferior to that for docetaxel monotherapy, whereas in a study performed by Maruyama *et al* (2008), gefitinib monotherapy did not result in non-inferior survival (Kim *et al*, 2008; Maruyama *et al*, 2008). Gefitinib therapy elicits extraordinary responses in patients who are women, patients who have never smoked, patients with adenocarcinomas, patients of Asian origin, and patients with an EGFR mutation (Lynch *et al*, 2004; Paez *et al*, 2004; Thatcher *et al*, 2005; Park and Goto, 2006).

Recently, phase III studies in patients with these clinical backgrounds or molecular mutations demonstrated that gefitinib monotherapy improved progression-free survival (PFS) as compared with platinum-doublet chemotherapy (Mok *et al*, 2009a; Maemondo *et al*, 2010; Mitsudomi *et al*, 2010). Therefore, gefitinib monotherapy has become a standard therapy for the treatment of advanced NSCLC. Unfortunately, even patients who show an initial response to gefitinib may eventually develop an acquired resistance to gefitinib. This happens, almost without exception, after varying periods of time. Two major mechanistic explanations have thus far been identified for acquired gefitinib resistance in NSCLC patients with an EGFR mutation. These include a second site EGFR mutation (T790M) and *MET* amplification (Kobayashi *et al*, 2005; Kwak *et al*, 2005; Pao *et al*, 2005; Engelman *et al*, 2007b).

Irinotecan (CPT-11) is a water-soluble derivative of camptothecin that inhibits DNA topoisomerase I (Kawato *et al*, 1991). In a phase III study comparing therapy with vindesine (VDS)+CDDP to therapy with CPT-11 alone or to therapy with CPT-11+cisplatin (CDDP), no significant difference was observed between the overall survival (OS) achieved with CPT-11+CDDP and with VDS+CDDP, and between the OS achieved with CPT-11 alone and with VDS+CDDP. In subgroup analyses, OSs of CPT-11+CDDP- and CPT-11-treated patients were superior to VDS+CDDP-treated patients with stage IV disease (Negoro *et al*, 2003). Based on this result, CPT-11 is thought to be a key drug for NSCLC treatment.

Preclinical studies have shown that the combination of CPT-11 and gefitinib has a synergistic beneficial effect in various tumour cell lines (Koizumi *et al*, 2004; Stewart *et al*, 2004; Shimoyama *et al*, 2006). Concerning NSCLC, the combination of CPT-11 and gefitinib is synergistic in EGFR wild-type cell line, mutant cell line, and gefitinib-resistant cell line. Furthermore, gefitinib-resistant NSCLC cells are more sensitive to CPT-11 than parental cells are, and sequential administration of CPT-11 and gefitinib has more remarkable beneficial effects than concurrent administration of both (Shimoyama *et al*, 2006). Based on this evidence, we conducted a phase I study to evaluate the combination of CPT-11 and gefitinib as a therapeutic option for NSCLC patients with progressive disease (PD) previously treated with gefitinib alone.

## Methods

### Study design

This phase I study was conducted in patients with advanced NSCLC previously treated with gefitinib. The primary objective of this study was to determine the maximum tolerated dose (MTD) of CPT-11 that could be administered in combination with gefitinib. Secondary objectives included determination of dose-limiting toxicities (DLTs) and a dosing recommendation for a phase II trial involving administration of CPT-11 plus gefitinib. Study treatment was provided until disease progression or unacceptable toxicities occurred.

### Treatment schedule

The patients were treated with CPT-11 on days 1 and 15. Gefitinib was administered daily from day 2 until day 28 in cycle 1 and from day 1 until day 28 after cycle 2. This treatment cycle was repeated every 4 weeks. The treatment was continued until disease progression. CPT-11 diluted in 250 ml of 5% glucose was administered intravenously over 90 min. Prophylactic antiemetic therapy, consisting of granisetron (40 mg kg^−1^) and dexamethasone (8 mg per body weight), was routinely prescribed.

### Treatment modification

CPT-11 was not administered if any of the following toxicities were noted on day 1 or 15: leucocytes <3000 mm^−3^, platelets <100 000 mm^−3^, serum creatinine >1.5 mg dl^−1^, total bilirubin >1.5 mg dl^−1^, aspartate aminotransferase (AST) or alanine aminotransferase (ALT) >3 times the upper limit of normal, grade 1 or higher infection, and grade 2 or higher diarrhoea. Gefitinib was not administered when grade 3 or higher rash or unacceptable toxicity occurred.

### Dose escalation

The gefitinib dose was fixed at 250 mg per body weight. The following dose levels of CPT-11 were administered: level 1, 50 mg m^−2^; level 2, 75 mg m^−2^; level 3, 100 mg m^−2^; level 4, 125 mg m^−2^; and level 5, 150 mg m^−2^. The dosing of CPT-11 was escalated in different patients at 25 mg m^−2^ increments with an upper limit of 150 mg m^−2^, which is the recommended biweekly single agent dose of CPT-11 in Japan. The MTD of CPT-11 was defined as the dose at which at least two of three or three of six patients developed DLT during the first cycle of treatment. Intrapatient dose escalation was not permitted. If MTD was not reached on level 5, level 5 was defined as the recommended dose for this study. Six to nine additional patients were treated at the recommended phase II dosing to confirm tolerability and response of this combination therapy.

### Eligibility criteria

The eligibility criteria for enrolment in this study were as follows: histologically confirmed NSCLC, age range of 20–74 years, progression of disease even after gefitinib treatment, one or more previous chemotherapy regimens, Eastern Cooperative Oncology Group performance status of 0–2, life expectancy of at least 3 months, adequate organ function (leucocyte count ⩾4000 mm^−3^, haemoglobin level ⩾9.0 g dl^−1^, platelet count ⩾100 000 mm^−3^, serum creatinine level ⩽1.5 mg dl^−1^, total bilirubin level ⩽1.5 mg dl^−1^, AST and ALT levels ⩽3 times the upper limit of the normal range, and arterial partial pressure of oxygen [PaO_2_] ⩾60.0 torr). Patients were excluded from the trial for any of the following reasons: uncontrolled malignant pleural or pericardial effusion, a concomitant serious illness contraindicating chemotherapy, history of pneumonitis during previous gefitinib therapy, pregnancy, or breast feeding. All patients provided written informed consent. The study protocol was approved by the institutional ethics committee of each of the participating institutions.

### Assessment

Toxicities were monitored, graded, and recorded according to the National Cancer Institute Common Toxicity Criteria version 2.0. DLT was defined as follows: grade 4 haematologic toxicities excluding neutropenia, grade 4 neutropenia lasting 4 days or longer, grade 3 or greater febrile neutropenia, and grade 3 or greater non-haematological toxicity excluding nausea, vomiting, and general fatigue. Efficacy was assessed by a physician on the basis of antitumour effect, according to the RECIST version 1.0. The response was confirmed for at least 4 weeks (for a complete or partial response (PR)) or 6 weeks (for stable disease (SD)) after it was first documented. Survival distribution was estimated by the Kaplan–Meier method.

## Results

### Patient characteristics

From December 2003 to March 2008, 27 patients were enroled in this study. Patient characteristics are summarised in Table 1. The median age of patients entering this study was 60 years. The median performance status was 1. Approximately half of the patients were male, and adenocarcinoma was of a major histologic type. Twelve patients (44.4%) were non-smokers. Before entering this study, all eligible patients had received various chemotherapies. Approximately 50% of the patients had received three chemotherapy regimens. Importantly, all studied patients had received gefitinib, and most patients had received gefitinib as a second-line therapy. Previous gefitinib therapy resulted in 21 patients (77.8%) with PR, 2 patients (7.4%) with SD, and 4 patients (14.8%) with PD. Five patients had acquired resistance to initial gefitinib treatment according to the criteria proposed by Jackman *et al* (2010). One patient had been administered level 2 doses; 2 patients, level 3 doses; and 2 patients, level 5 doses. All five patients achieved PR with initial gefitinib treatment. Three patients developed PD within 1 month of initial gefitinib treatment; these patients might have had primary resistance to initial gefitinib treatment. Two of these patients had been administered level 1 doses, and 1 patient had been administered level 2 doses.

Dosing information is listed in Table 2. In this study, a total of 87 cycles of therapy were given. The number of treatment cycles administered per patient ranged from 1 to 10 (median, 4 cycles).

### Safety

Toxicities were evaluated in 27 patients. The DLTs were observed in only 2 patients at level 3 dosing. We defined level 5 as the recommended dose for this study. We added 9 patients at level 5 to confirm tolerability and the response of this combination therapy. Toxicities are summarised in Table 3. The major haematologic toxicities included neutropenia and leucopoenia with dose-dependent occurrence. However, only 2 grade 4 cases of neutropenia were noted at level 5. No patients experienced febrile neutropenia. The major non-hematologic toxicities were nausea, vomiting, and diarrhoea. There was no grade 3 or 4 non-haematologic toxicity except diarrhoea. Grade 3 diarrhoea was observed in 4 patients. No patients experienced pneumonitis. There were no treatment-related deaths.

### Efficacy

Twenty-six patients (96.3%) were analysed for response to therapy; 7 patients had PR, 11 patients had SD, and 8 patients had PD (Table 4). The disease control rate (DCR) and response rate (RR) were 69.2% and 26.9%, respectively. From the 12 patients who received level 5 doses (recommended phase II dose), 5 patients achieved PR, 4 patients had SD, and 3 patients had PD. The DCR and RR at level 5 were 75.0% and 41.7%, respectively. From the five patients with acquired resistance to initial gefitinib treatment, one patient achieved PR, two patients had SD, and two patients had PD. From the three patients with primary resistance to initial gefitinib treatment, one patient had SD and two patients had PD. The median time to treatment failure was 57 days (95% confidence interval (CI), 32–82 days); median PFS, 70 days (95% CI, 38–102 days); median OS, 244 days (95% CI, 185–303 days); and 1-year survival rate, 32.6%.

### EGFR mutation analysis

EGFR mutations were analysed in 10 of 27 patients by direct sequencing of paraffin-embedded tumour samples extracted before initiation of gefitinib therapy. The EGFR mutations were detected in 4 of 10 patients. In-frame deletions within exon 19 were detected in three patients, and a mutation of L858R was detected within exon 21 in one patient. In the patients with EGFR mutation, two patients had PR, one patient had SD, and one patient had PD. In the other six patients with wild-type EGFR, one patient had PR, one patient had SD, three patients had PD, and one patient was not evaluable. The data are summarised in Table 5. The patients did not undergo re-biopsy after initial gefitinib treatment.

## Discussion

Our study showed that combining CPT-11 and gefitinib is feasible without adverse toxicity. The MTD was not determined, and full dose CPT-11 and gefitinib could be combined. Diarrhoea was one of the major non-hematologic toxicities present in the study. Diarrhoea occurred in 22 patients (81.5%), but grade 3 diarrhoea was observed in only 4 patients (15%). Diarrhoea is a major common toxicity of both drugs. Frequency of grade 3 diarrhoea was 21% in a phase II study of CPT-11 monotherapy (Fukuoka *et al*, 1992), and 0–1% in phase II studies of gefitinib monotherapy (Fukuoka *et al*, 2003; Kris *et al*, 2003). Based on these studies, the frequency of diarrhoea was not increased upon CPT-11 and gefitinib combined dosing.

The effect of combination therapy with gefitinib and cytotoxic chemotherapy has already been evaluated for previously untreated advanced NSCLCs. Additionally, two randomised phase III studies evaluated the addition of gefitinib to standard platinum-doublet chemotherapy and found no significant improvement in OS (Giaccone *et al*, 2004; Herbst *et al*, 2004). However, recently, a randomised phase II study showed that sequential administration of EGFR-TKI, following chemotherapy, led to a significant improvement in PFS versus administration of chemotherapy alone in unselected, previously untreated patients with advanced NSCLC (Mok *et al*, 2009b).

The DCR and RR were 69.2% and 26.9%, respectively, in all patients in our trial, and 75.0% and 41.7% at level 5, respectively. These DCR and RR were very encouraging, especially as patients were resistant to gefitinib. In the two published Japanese phase II studies of CPT-11, in which CPT-11 was administered to previously treated NSCLC patients, RRs were 0% and 13.6%, respectively (Nakai *et al*, 1991; Negoro *et al*, 1991). The efficacy of CPT-11 for treating patients with an EGFR mutation is not known; therefore, it is possible that CPT-11 is effective for patients with an EGFR mutation. However, we hypothesised that CPT-11 and gefitinib would have a synergistic and beneficial effect clinically. To explain this synergistic effect, three biological mechanisms should be considered.

The first mechanism involves data reported in a study by Ohtsuka *et al* (2010). Resistance to EGFR-TKI is associated with the downregulation of ABCG2 expression. Additionally, EGFR-TKI-resistant cell lines with concomitant downregulation of ABCG2 expression have high sensitivity to a topoisomerase I inhibitor alone or in combination with EGFR-TKI when compared with cell lines with normal ABCG2 expression. Second, gefitinib-resistant cells have a high sensitivity to SN-38, a metabolite of the camptothecin derivative CPT-11. In our original data, the half maximal inhibitory concentration values for SN-38 were significantly lower in gefitinib-resistant cells than in gefitinib-sensitive parental cells. We observed an increase in topoisomerase I mRNA expression in gefitinib-resistant cells (unpublished data). Third, the combination of topotecan and gefitinib has been reported to have a synergistic effect in a topotecan-resistant cell line (Yang *et al*, 2005); topotecan is a topoisomerase I inhibitor. Collectively, these data suggest that overall sensitivity to CPT-11 should increase in our patients. Therefore, CPT-11 and gefitinib should have a synergistic beneficial effect in NSCLC patients with acquired resistance to gefitinib.

The mechanistic reasons behind the resistance to EGFR TKI are different, probably involving T790M secondary mutation and *MET* amplification. Recently, some irreversible EGFR-TKIs and MET inhibitors have shown antitumour activity in patients resistant to gefitinib or erlotinib in pre-clinical studies (Kwak *et al*, 2005; Gendreau *et al*, 2007; Engelman *et al*, 2007a; Li *et al*, 2008; Tang *et al*, 2008). At least three irreversible EGFR-TKIs (neratinib (HKI-272), XL647, and PF-00299804) were used in phase II studies of NSCLC patients with acquired resistance to gefitinib or erlotinib (Rizvi *et al*, 2008; Janne *et al*, 2009; Sequist *et al*, 2010). The RRs were 3.4% (EGFR mutant) and 0% (EGFR wild-type) in neratinib, 4.3% in XL647, and 7.0% in PF-00299804. The RRs of our combination of gefitinib and CPT-11 were similar to those for irreversible EGFR-TKIs treatment. Therefore, we think that this combination can be used as a treatment option for patients failing gefitinib monotherapy.

In conclusion, the combination of CPT-11 and gefitinib was well tolerated and potentially therapeutic for NSCLC patients no longer responding to gefitinib monotherapy. Larger phase II studies are required to evaluate the efficacy of this combination therapy for NSCLC patients with PD no longer responding to gefitinib treatment.

## Figures and Tables

**Table 1:** Patient characteristics

**Characteristics**	**Number of patients**
Patients	27
	
*Gender*	
Male	14
Female	13
	
*Age (years)*	
Mean	60
Range	45–75
	
*Performance status*	
0	2
1	17
2	8
	
*Smoking status*	
Current	7
Former	8
Never	12
	
*Histology*	
Adenocarcinoma	25
Squamous cell carcinoma	1
Large cell carcinoma	1
	
*EGFR mutation status*	
Positive	4
Negative	6
Unknown	17
	
*Number of previous chemotherapy regimens*	
2	3
3	15
4	8
5	1
	
*Previous gefitinib response*	
PR	21
SD	2
PD	4

Abbreviations: EGFR=epidermal growth factor receptor; PR=partial response; SD=stable disease; PD=progressive disease.

**Table 2:** Dose-limiting toxicities

		**Gefitinib**	**Number of patients**		**Type of DLT**
**Level**	**CPT-11 (mg m^−2^)**	**(mg per body weight)**	**Evaluable**	**DLT**	**(number of patients)**
Level 1	50	250	3	0	
Level 2	75	250	3	0	
Level 3	100	250	6	2	Diarrhoea (2)
Level 4	125	250	3	0	
Level 5	150	250	3	0	

Abbreviations: CPT-11=irinotecan; DLT=dose-limiting toxicity.

**Table 3:** Toxicities in (a) first cycle; (b) all cycles

*(a)*																				
	**Level 1 (*N*=3)**				**Level 2 (*N*=3)**				**Level 3 (*N*=6)**				**Level 4 (*N*=3)**				**Level 5 (*N*=12)**			
**NCI-CTC grade**	**1**	**2**	**3**	**4**	**1**	**2**	**3**	**4**	**1**	**2**	**3**	**4**	**1**	**2**	**3**	**4**	**1**	**2**	**3**	**4**
Leucocytes	0	0	0	0	0	0	0	0	1	3	0	0	2	0	0	0	4	3	2	0
Neutrophils	0	0	0	0	0	0	0	0	3	0	1	0	1	0	0	0	2	3	3	1
Haemoglobin	3	0	0	0	1	1	0	0	3	1	0	0	2	0	0	0	5	2	1	0
Platelets	0	0	0	0	0	0	0	0	0	0	0	0	0	0	0	0	2	0	0	0
General fatigue	0	0	0	0	0	1	0	0	3	0	0	0	1	0	0	0	3	1	0	0
Rash	3	0	0	0	3	0	0	0	4	0	0	0	1	0	0	0	4	1	0	0
Nausea/vomiting	1	0	0	0	1	1	0	0	4	0	0	0	2	1	0	0	8	4	0	0
Diarrhoea	1	1	0	0	2	0	0	0	2	0	2	0	1	2	0	0	6	3	2	0
Stomatitis	0	0	0	0	1	0	0	0	1	0	0	0	1	0	0	0	0	3	0	0
AST, ALT	1	0	0	0	2	0	0	0	1	2	0	0	1	0	0	0	5	2	0	0
Total bilirubin	0	0	0	0	0	0	0	0	1	0	0	0	1	0	0	0	3	1	0	0
Creatinine	0	0	0	0	0	0	0	0	0	0	0	0	0	0	0	0	2	0	0	0

*(b)*											
	**Level 1–4 (*N*=15)**					**Level 5 (*N*=12)**					**Total (*N*=27)**
**NCI-CTC grade**	**1**	**2**	**3**	**4**	**3–4(%)**	**1**	**2**	**3**	**4**	**3–4(%)**	**3–4(%)**
Leucocytes	3	3	1	0	7	2	5	3	0	25	15
Neutrophils	2	2	3	0	20	2	1	5	2	58	37
Haemoglobin	9	5	0	0	0	7	3	1	0	8	4
Platelets	0	0	0	0	0	2	0	0	0	0	0
General fatigue	4	1	0	0	0	3	1	0	0	0	0
Rash	9	0	0	0	0	4	1	0	0	0	0
Nausea/Vomiting	8	2	0	0	0	8	4	0	0	0	0
Diarrhoea	5	4	2	0	13	5	4	2	0	17	15
Stomatitis	4	0	0	0	0	1	3	0	0	0	0
AST, ALT	5	2	0	0	0	5	2	0	0	0	0
Total bilirubin	2	0	0	0	0	4	2	0	0	0	0
Creatinine	0	0	0	0	0	4	0	0	0	0	0

Abbreviations: N=number of patients; AST=aspartate aminotransferase; ALT=alanine aminotransferase.

**Table 4:** Response

	**Level 1 (*N*=3)**	**Level 2 (*N*=3)**	**Level 3 (*N*=6)**	**Level 4 (*N*=3)**	**Level 5 (*N*=12)**	**Total (*N*=27)**
PR	0	0	0	2	5	7
SD	2	1	4	0	4	11
PD	1	2	2	0	3	8
NE	0	0	0	1	0	1

Abbreviations: N=number of patients; PR=partial response; SD=stable disease; PD=progressive disease; NE=not evaluable.

**Table 5:** Biomarker analysis and clinical outcome

**Patient number**	**Gender**	**Age**	**Histology**	**Previous gefitinib response**	**EGFR mutation status**	**Response to CPT-11 and gefitinib**
L1-2	Male	54	Squamous	PD	Wild	PD
L2-3	Female	55	Adeno	PD	Wild	PD
L3-5	Female	75	Adeno	PR	Wild	SD
L4-2	Female	57	Adeno	PR	Wild	NE
L5-1	Female	58	Adeno	PR	L858R	SD
L5-2	Male	63	Adeno	PR	Wild	PR
L5-3	Male	75	Adeno	PD	Wild	PD
L5-4	Male	64	Adeno	PR	Deletion	PR
L5-9	Female	64	Adeno	PR	Deletion	PR
L5-12	Female	48	Adeno	PR	Deletion	PD

Abbreviations: CPT-11=irinotecan; EGFR=epidermal growth factor receptor; Squamous=squamous cell carcinoma; Adeno=adenocarcinoma; PR=partial response; SD=stable disease; PD=progressive disease; NE=not evaluable; Wild=wild-type; L858R=L858R within exon 21; Deletion=in-frame deletions within exon 19.
